# Extraction of Human Limbs Based on Micro-Doppler-Range Trajectories Using Wideband Interferometric Radar

**DOI:** 10.3390/s23177544

**Published:** 2023-08-30

**Authors:** Xianxian He, Yunhua Zhang, Xiao Dong

**Affiliations:** 1 CAS Key Laboratory of Microwave Remote Sensing, National Space Science Center, Chinese Academy of Sciences, Beijing 100190, China; hexianxian19@mails.ucas.ac.cn (X.H.); dongxiao@mirslab.cn (X.D.); 2 School of Electronic, Electrical, and Communication Engineering, University of Chinese Academy of Sciences, Beijing 100049, China

**Keywords:** human micromotion, human limbs extraction, components separation, interferometric radar, micro-Doppler (mD), micro-Doppler signature (mDS), micro-Doppler-Range signature (mDRS)

## Abstract

In this paper, we propose to extract the motions of different human limbs by using interferometric radar based on the micro-Doppler-Range signature (mDRS). As we know, accurate extraction of human limbs in motion has great potential for improving the radar performance on human motion detection. Because the motions of human limbs usually overlap in the time-Doppler plane, it is extremely hard to separate human limbs without other information such as the range or the angle. In addition, it is also difficult to identify which part of the body each signal component belongs to. In this work, the overlaps of multiple components can be solved, and the motions from different limbs can be extracted and classified as well based on the extracted micro-Doppler-Range trajectories (MDRTs) along with a proposed three-dimensional constant false alarm (3D-CFAR) detection. Three experiments are conducted with three different people on typical human motions using a 77 GHz radar board of 4 GHz bandwidth, and the results are validated by the measurements of a Kinect sensor. All three experiments were repeatedly conducted for three different people of different heights to test the repeatability and robust of the proposed approach, and the results met our expectations very well.

## 1. Introduction

Using radar to detect human motion for classification and recognition has attracted significant attention in recent years [1,2,3]. Recent works have been focused on human skeletal posture estimation [4,5,6], human parsing [7], and 3D body mesh estimation [8,9] based on millimeter-wave MIMO radar. Except for the potential applications for human–computer interaction and gait recognition [10,11], there is a great potential to monitor human motions without privacy invasion for the physical health care of elderly people and patients [12,13]. Human radar micro-Doppler signatures (mDS), as a significant feature that can be used to classify and recognize human motions, contain the time-varying velocity information of human limbs [14]. In human–computer interaction applications, the device should conjecture the intention of human behavior before the response. In automated driving applications, the driving-assistant system needs to judge the intention of pedestrian before taking actions. The accurate extraction of the motion data of different limbs allows for the quantitative interpretation of human motion, which helps to understand the behavior. Compared to the classification without separation via preprocessing, the separated mDS of different body parts can significantly improve the classification accuracy [15]. Traditionally, the mDS is extracted in the time-Doppler domain [16], which is inadequate for discriminating human limbs because only the velocity information is used. To extract motion data of human limbs and improve the radar performance on detecting human motions, more information such as range or angle should be involved to supplement the mDS.

Raj et al. proposed approaches to infer and decompose the mDS into several signals corresponding to the major human joints, which was validated by using simulated mDSs corresponding to simple motions [17]. An algorithm based on principle component analysis was proposed for decomposing the mDSs of human motions in [18], and it was also validated just by simulation. In [15,19], the authors proposed a method based on short-time fractional Fourier transform to separate the mDS into two parts, i.e., one for torso and the other for limbs. This method was verified by both simulation and real experiments, but it still could not separate the components of different limbs. In addition, the method based on parametric sparse time-frequency representation was proposed in [20], which performed well on simulation data and was validated by simulation only.

Algorithms using both the mDS and the range information to decompose the motion data were proposed in [21,22]. The algorithm of [21] was verified by simulation, while that of [22] was validated against practical experiments. These methods demonstrated better performance than traditional methods in the mDS domain by decomposing motion components in the time-range-Doppler domain. However, all these methods are based on single channel radar lacking the angle information of the motion components in either the time-Doppler or time-range-Doppler domain. Therefore, although the above works are able to estimate and decompose human motion components through signal processing, the motion components are difficult to classify into the corresponding limbs.

Interferometric radar has been widely used in human detection, tracking, and imaging because the direction of arrival (DOA) of the target can be obtained by utilizing the interferometric phase. Lin and Ling [23,24,25] proposed to use very low-complexity continuous wave interferometric Doppler radar for human imaging and multiple human mover tracking, in their approaches, the divisibility of the multiple movers’ Doppler frequencies was used to obtain the DOA information of the movers. Ram et al. further utilized more receivers for achieving better tracking performance for multiple humans [26], and the authors also explored through-wall multiple human subjects tracking by using a four-element radar [27]. Later, Sakamoto and Saho et al. applied ultra-wideband (UWB) interferometric radar to separating and imaging multiple human targets based on their Doppler frequencies and their range bins [28,29,30]. The above works have demonstrated the advantages of using interferometric radar on the separating and imaging of multiple human targets and have shown potential advantages in extracting human limbs as well. But compared with separating different human subjects, there are fewer works reported up to now on separating human limbs by using interferometric radar, as shown in Table 1.

Recently, Steinhauser and Held et al. proposed a radar-based method to separate different body parts of a pedestrian, utilizing range, Doppler frequency, and azimuth angle information for automotive safety applications [31]. However, the performance of this method is limited by the pedestrian’s walking states, because the torso, legs, and hands are iteratively extracted according to their intensities (i.e., the torso with the highest intensity and hands with the smallest intensity). If a human stands in a place and only swings arms (or marking on time without hand motions), the Doppler frequencies of the torso and feet (or hands) will not be differentiable, and their intensities of radar echo will be added together making it difficult to judge whether the hands or feet are in motion due to the lack of elevation information. As mentioned in [31], additional elevation information is helpful for extracting different parts of human body. Further, Held and Steinhauser et al. proposed a method for pedestrian tracking by using a new-generation automotive radar sensor which has 16 receive antennas, which were divided into two subarrays with 12 and 5 receive antennas, respectively [32], to obtain both the elevation information and the azimuth information, and the elevation information was used to extract the reflection points caused by moving legs for pedestrian tracking.

In this paper, we propose an interferometric radar approach to extract human limbs from micro-Doppler-Range trajectories (MDRTs). The time-Doppler-Range characteristics are used to represent the micromotions of a moving target aiming to solve the overlapping problem existing in the time-Doppler domain, after which, the interferometric phase is utilized to acquire the angle information. Benefitting from the angle information, the elevation and azimuth positions can be fixed, and thus different human limbs can be classified according to the micro-Doppler-Range signatures (mDRSs), i.e., different limbs can thus be separated and extracted.

The remainder of this paper is arranged as follows. Section 2 briefly presents the fundamentals of mDS and radar interferometry. Based on the mDRS, the method for limbs extraction by using wideband interferometric radar is developed in Section 3. Practical experiments are described with the results analyzed in Section 4, and finally the paper is concluded in Section 5.

## 2. Radar mDS and Interferometry

### 2.1. Human Micro-Doppler Signature

Micromotions of a target or a structure, such as human arms swinging when walking, induce the well-known micro-Doppler phenomenon in radar detection. The mDS reflects the motion kinematics of a target in the time-Doppler domain, which can be obtained by taking the time-frequency analysis on radar echo signals [16].

A human is one of the representative targets of micromotion signatures. When a human walks towards radar, their limbs, such as arms and legs, all exhibit micromotions with different velocities. So, the received radar signal containing the information of all micromotions can be expressed after demodulation as

(1) $$s_{r}\left(t\right)=\sum_{i}A_{i}exp\left[j2\pi f_{di}\left(t\right)t\right],$$

where $A_{i}$ is the amplitude, and $f_{di}\left(t\right)$ is the micro-Doppler frequency corresponding to the micromotion component (MMC) i. By conducting Short-Time Fourier Transform (STFT) on $s_{r}\left(t\right)$, we obtain

(2) $$S\left(t,f\right)=\int_{\tau }s_{r}\left(\tau \right)w\left(\tau -t\right)exp\left(-j2\pi f\tau \right)d\tau ,$$

where $w\left(t\right)$ is the time window function; the micromotions can thus be characterized in the time-frequency plane, and the mDS can be obtained.

**Figure 1:**
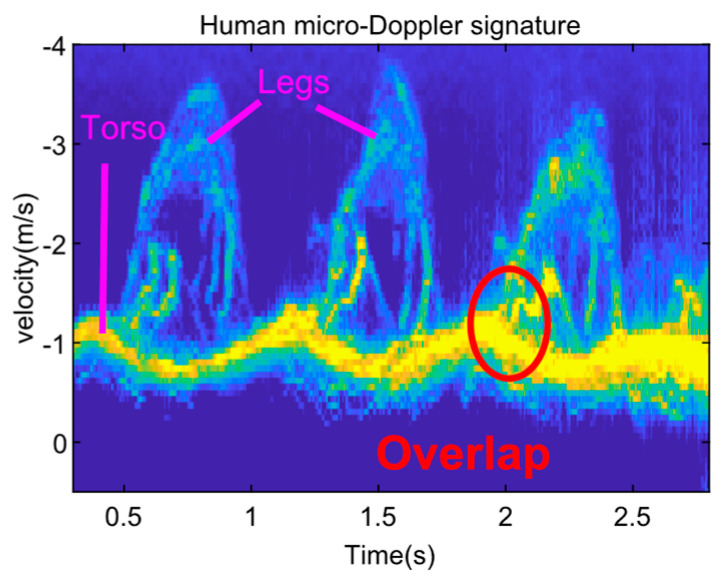
mDS of a pedestrian with no arm swinging, the echo strength is represented by color, e.g. the red circled part denotes stronger echo.

Figure 1 shows the mDS of a human walking towards a Ku-band single channel radar with no arm swinging. There are three major m-D components induced by the torso, left leg and right leg, respectively. As shown in Figure 1, the first component is induced by the torso exhibiting the strongest intensity, whose velocity exhibits a pseudo-periodic oscillation between about −0.5 m/s and −1.5 m/s. The remaining two components are induced by legs exhibiting the largest radial velocity about −3.5 m/s in peak.

However, when the radar echo data of Figure 1 are used to extract the micromotions of limbs, the following problems are encountered.

1. The velocities of the torso, legs, and arms are hard to identify and interpret even by professionals without prior knowledge about human motions.
2. The multiple m-D components including the torso, legs, and arms are overlapped with each other in the mDS. Thus, it is hard to extract human limbs accurately only based on mDS. As we will show in Section 3, the overlapping problem can be solved by incorporating the range information into mDS.
3. Although the velocity components may be separated from the mDS by signal decomposition [17,18], it is still difficult to identify which limbs induced them. As shown later, this problem can be solved by utilizing the interferometric phases obtained by interferometric radar.

### 2.2. Radar Interferometry

**Figure 2:**
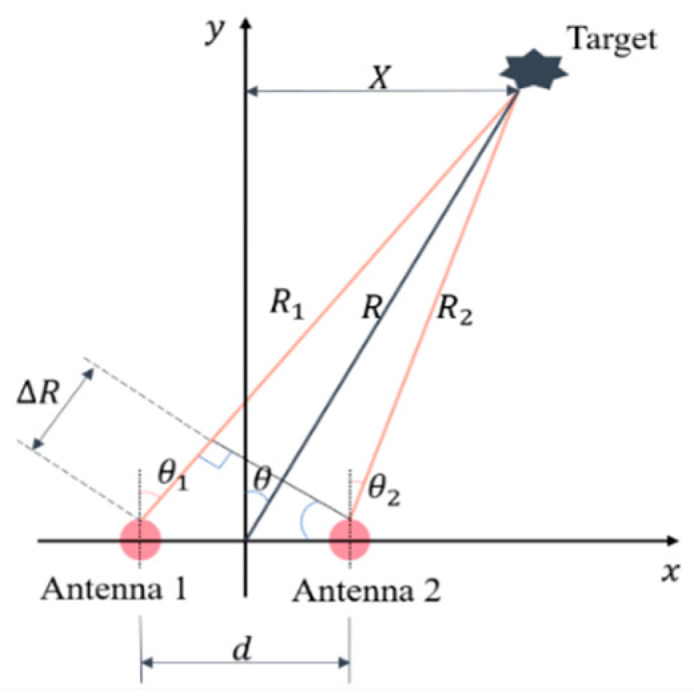
Interferometric radar with two antennas.

Let us consider an interferometric radar composed of two antennas as shown in Figure 2, whose positions are $\left(-\frac{d}{2},0\right)$ and $\left(\frac{d}{2},0\right)$, respectively, i.e., the baseline length is d. The distance between the target and the middle of two antennas is R, and R≫d. Thus, the difference between $\theta_{1}$ and $\theta_{2}$ is negligible, i.e.,$\theta_{1}\approx \theta_{2}=\theta$, and $R_{1}-R_{2}=∆R\approx dsin\theta .$

Assuming Antenna1 is used for transmitting, and both antennas are used for receiving, the measured phase difference ∆ϕ of these two antennas can be expressed as

(3) $$∆\phi =\frac{2\pi ∆R}{\lambda }=\frac{2\pi dsin\theta }{\lambda },$$

where λ is the wavelength of the central frequency of the transmitted signal. Therefore, the azimuth angle θ and position X can be estimated by

(4) $$\theta =\sin^{-1}\frac{∆\phi \lambda }{2\pi d},$$

(5) $$X=Rsin\theta =\frac{∆\phi \lambda R}{2\pi d}.$$

Interferometric radar can discriminate multiple targets by estimating their angles, referring to the normal of the baseline according to the measured interferometric phases [33], and the angle information can then be used to extract the motion components induced by different human limbs. Let us suppose a human is walking towards a radar, whose arms and legs are at different elevation positions and whose left limbs and right limbs are at different azimuth positions. It is possible to discriminate and extract the different limbs by using an interferometric radar. However, if the limbs have the same radial velocity at the same time, different m-D components or different micromotions will overlap with each other as shown in Figure 1, In this case, if we just utilize the interferometric phase alone, we still cannot extract the MMCs accurately based on the human mDSs. In the next section, we show that different MMCs can be well separated and extracted by incorporating range information to mDS, utilizing the interferometric information together.

## 3. Micro-Doppler-Range Trajectory Extraction

According to the fact that human mDS can provide an aggregation of the time–velocity distribution of human limbs, the precondition for separating the limbs from mDS using interferometric radar is that the MMCs are not overlapped. However, as described above, the overlapping problem is unavoidable in human mDS. To solve the overlaps and extract human limbs accurately, we propose to use the micro-Doppler-Range signature (mDRS), which contains both range and velocity information.

### 3.1. Human mDRS

Just as the mDS can be presented in the time-Doppler plane, the micro-Range Signature (mRS) induced by micromotions of limbs can also be presented in the time-Range plane if high resolution range information is available by using wideband radar [22]. However, no matter the mDS or the mRS is used, overlaps between human limbs are unavoidable.

**Figure 3:**
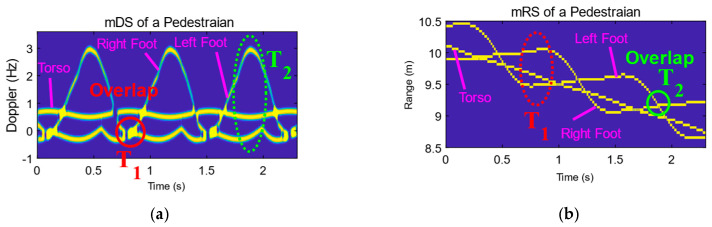

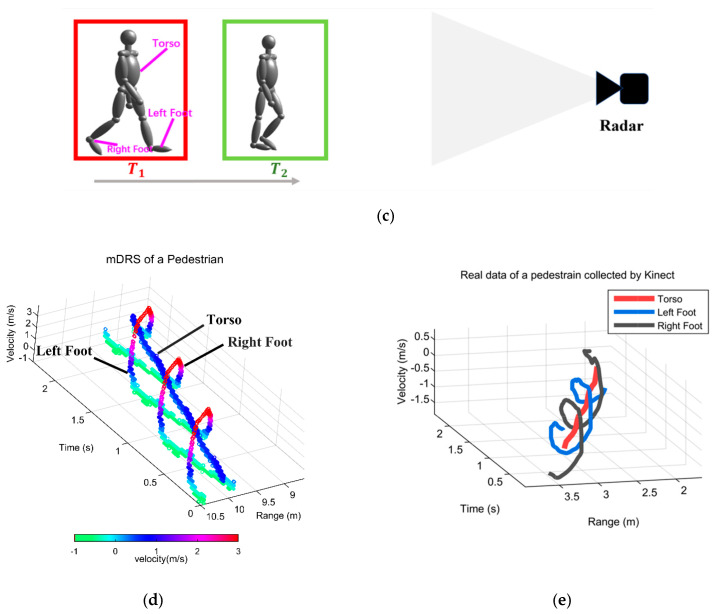
Radar simulation and real Kinect data of a pedestrian. (**a**) Simulated mDS. (**b**) Simulated mRS. (**c**) Two walking states. (**d**) Simulated mDRS. (**e**) Real data collected by Kinect.

Figure 3 shows the simulated mDS, mRS, mDRS, and real Kinect data of a pedestrian. The simulation is conducted using the method proposed in [34]. To simplify the discussion, only the feet and torso are taken into consideration in this simulation. There are two overlaps, one is the mDS overlap, and the other is the mRS overlap.

1. mDS overlap. In Figure 3a, the circled part in red denotes the mDS overlap, which is labeled by $T_{1}$. They happen at the instants when both feet are on the ground. The red box in Figure 3c shows the corresponding diagram of the $T_{1}$ state.
  As the distances of the two feet from the radar are different in the $T_{1}$ state, there is no overlap for the mRS in Figure 3b, shown by the red dotted circle.
2. mRS overlap. The green solid circle marked as $T_{2}$ in Figure 3b denotes the mRS overlap. In this situation, both feet have the same distance relative to the radar. The green box in Figure 3c shows the diagram of the $T_{2}$ state.
  Although the two feet have the same range, their velocities are different, i.e., the standing foot is at zero velocity, while the other foot is at the maximum radial velocity within a gait cycle. As shown in Figure 3a, the mDSs of the two feet do not overlap with each other.

For a pedestrian, whose two feet usually either have the same velocity or have the same distance, either the mDS overlap or the mRS overlap is usually unavoidable. However, as shown in Figure 3a,b, when the two feet have the same velocity, their distances relative to radar are different, while when the two feet have the same distance, their velocities are different. This is to say that the overlapping problem can be well handled if both the velocity information and the distance information, i.e., the mDRS, are used.

Figure 3d presents the simulated mDRS of a pedestrian, where both the feet and the torso are well-separated without overlapping. This is true for swinging arms; although they are not shown here, the case is the same. Moreover, the real Kinect data of a pedestrian shown in Figure 3e also demonstrate that there are no overlapping problems when cooperating the range information with velocity information. Thus, if we take the mDRS to solve the overlapping problem, good results can be expected.

### 3.2. Interferometric Geometry and Retrieval of Positions

**Figure 4:**
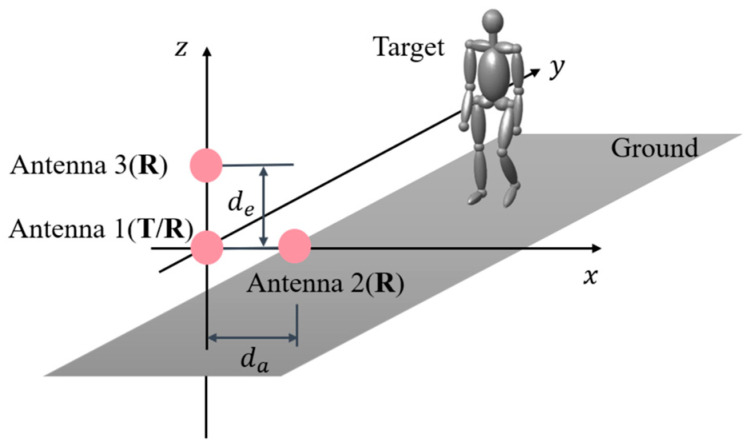
Observation geometry using an interferometric radar.

In this work, the simplest and widely used interferometric radar geometry formed by three antennas in L-shape [35,36] is adopted, where both the elevation interferometry and the azimuth interferometry are constructed as shown in Figure 4. Three antennas are located at (0,0,0), $\left(d_{a},0,0\right)$, and $\left(0,0,d_{e}\right),$ respectively, where Antenna1 both transmits and receives signals, while Antenna2 and Antenna3 only receive signals. This system constructs two orthogonal interferometric baselines, i.e., the horizontal and vertical baselines, which can be utilized to obtain the azimuth and elevation angle positions corresponding to different limbs of the pedestrian as shown in the following. Let us use $S_{1}(f,r,t)$, $S_{2}(f,r,t)$, and $S_{3}(f,r,t)$ to denote the mDRSs obtained from the received echoes by three antennas.

In real situations, the radar echoes are usually influenced by various interferences, e.g., the signal to noise ratio (SNR) and the background clutter. Therefore, the constant false alarm rate (CFAR) [37] method is usually used to detect a moving human target under a complex environment full of interference. Different from the traditional one-dimensional or two-dimensional CFAR, here, a three-dimensional CFAR (3D-CFAR) scheme is proposed to achieve a better performance, i.e., a 3D-CFAR window is applied to the data cube of the time-Doppler-range with the threshold T given by

(6) $$T=-\sigma_{m}^{2}\ln P,$$

where P is the false alarm probability set as a constant, and $\sigma_{m}^{2}$ is the interferometric power calculated as

(7) $$\begin{array}{c} \sigma_{m}^{2}=\frac{1}{N_{g}}\sum_{i=1}^{N_{g}}[{\left|S_{1}\left(f_{i},r_{i},t_{i}\right)\right|}^{2}+{\left|S_{2}\left(f_{i},r_{i},t_{i}\right)\right|}^{2}+{\left|S_{3}\left(f_{i},r_{i},t_{i}\right)\right|}^{2}], \end{array}$$

where $N_{g}$ is the number of guard cells in the CFAR window, and $S_{n}\left(f_{i},r_{i},t_{i}\right)(n=1,2,3)$ are the echoes received by three antennas corresponding to the ith guard cell in the Range-Doppler-Time cube. After the echoes from all the cells have been examined, those echoes whose powers exceed the corresponding thresholds proceed to the interferometric processing.

**Figure 5:**
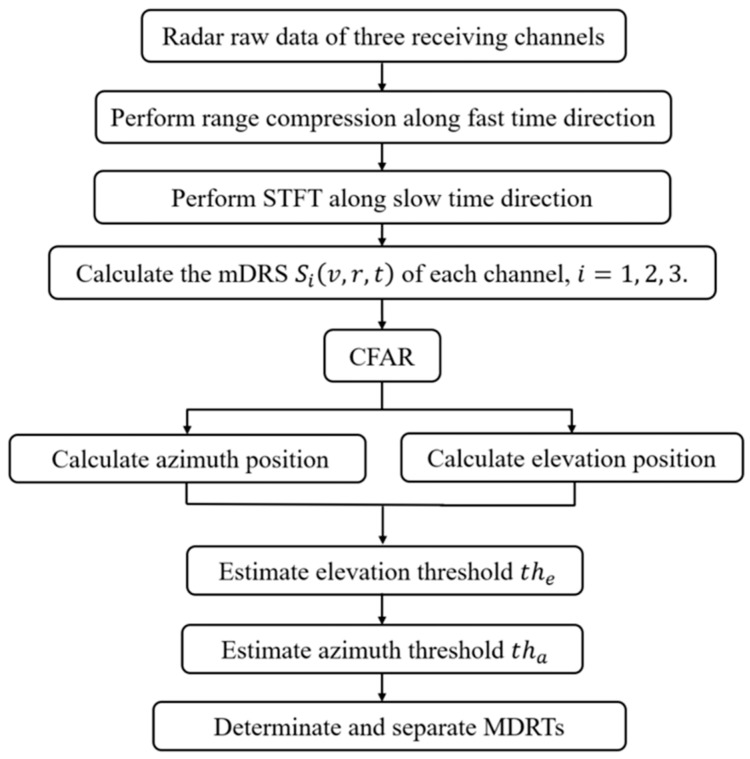
Flowchart for the extraction of MDRTs using interferometric radar.

It is worth noting that there are two improvements in this work compared to the traditional processing flow of point cloud generation [38]. As shown in Figure 5, one is the use of the sliding window STFT instead of the Doppler FFT in the slow time domain, and the sliding window processing reduces the time interval; the other is the use of 3D-CFAR instead of 2D-CFAR to realize better target detection for 3D (Time-Doppler-Range) data.

As shown in Figure 4, Antenna1 and Antenna2 form the azimuth interferometer, while Antenna1 and Antenna3 form the elevation interferometer. As a result, the azimuth phase difference $∆\phi_{a}$ and the elevation phase difference $∆\phi_{e}$ can be expressed, respectively, as

(8) $$∆\phi_{a}=∠[S_{1}\left(f,r,t\right)\cdot S_{2}^{*}\left(f,r,t\right)],$$

(9) $$∆\phi_{e}=∠[S_{1}\left(f,r,t\right)\cdot S_{3}^{*}\left(f,r,t\right)].$$

Having obtained the above interferometric phases, the azimuth position *X* and the elevation position *Z* can be calculated, respectively, via (5) as

(10) $$X=\frac{∆\phi_{a}\lambda R}{2\pi d_{a}},$$

(11) $$Z=\frac{∆\phi_{e}\lambda R}{2\pi d_{e}}.$$

Therefore, the corresponding spatial position corresponding to every mDRS component can be obtained; then, their attributions can be identified according to their spatial positions, and thus the MDRTs of human limbs can be extracted.

### 3.3. Extraction of the Micro-Doppler-Range Trajectory

In view of the characteristics of a pedestrian, the MMCs of arms and legs are adequate for interpreting human motion in most cases. Based on the above analyses, we summarize the method for extracting the mDRS trajectory by interferometric radar as the flowchart shown in Figure 5.

**Figure 6:**
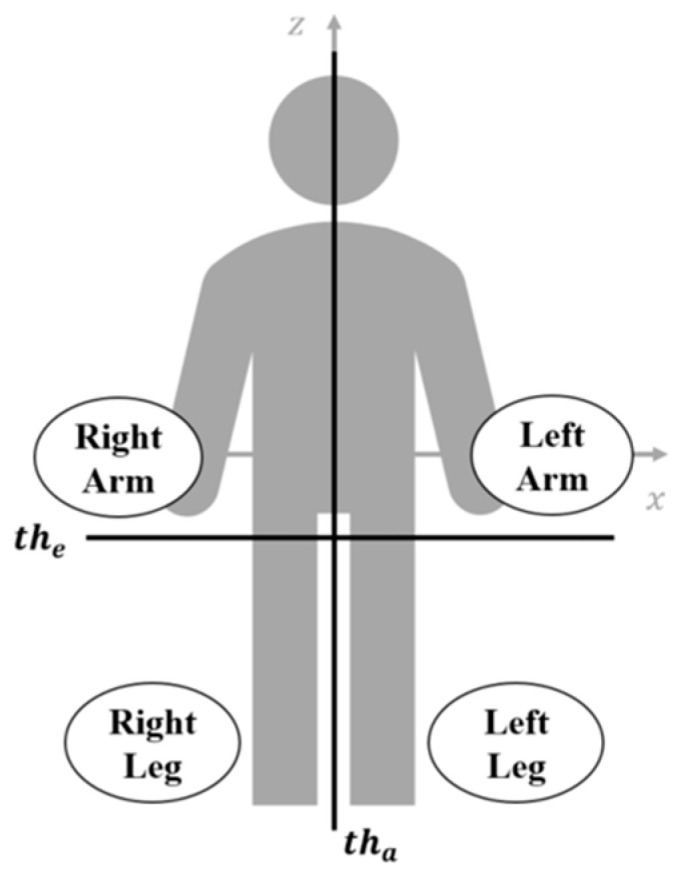
Sketch for threshold settings.

As shown in Figure 6, the elevation threshold $th_{e}$ is set to discriminate the motions of arms and legs according to their elevation positions, while the azimuth threshold $th_{a}$ is set to classify the left and right limbs. These two thresholds are utilized together to extract the MDRTs of limbs.

Because the torso is the strongest scatter of the human body, its height is used to define the elevation threshold. It is clear that the highest elevation positions above the torso should be the shoulders, while the lowest positions should be the hips. Therefore, we set the following elevation threshold to discriminate the upper body and the lower body,

(12) $$th_{e}=\frac{Z_{S}-Z_{G}}{2}+Z_{G}=\frac{Z_{S}+Z_{G}}{2},$$

where $Z_{S}$ and $Z_{G}$ are the elevation positions of the shoulder and ground (as shown in Figure 4), respectively.

The azimuth threshold $th_{a}$ can also be obtained from the echo data. For a walking human, there is always a foot standing on the ground without introducing micro-Doppler, while the upper body keeps moving. Therefore, we take the azimuthal average of the upper body as the azimuth threshold $th_{a}$. As shown in Figure 6, the azimuth threshold $th_{a}$ is the azimuth center of the torso. Thus, we can obtain the azimuth threshold $th_{a}$ by estimating the azimuth center of the torso

(13) $$th_{a}=\frac{1}{N}\sum_{i=1}^{N}X\left(k_{i}\right),\;such\;that\;Z(k_{i})\geq th_{e},\forall k_{i}\in (k_{1},k_{2},\ldots ,k_{N}),$$

where $k_{1},k_{2},\ldots ,k_{N}$ are the indexes of the echo data from the upper body, i.e., their elevation positions are higher than the elevation threshold $th_{e}$.

The procedure for setting thresholds includes the following four steps:

(1) Select the strongest scatter of the echo data at each moment and take the highest elevation position as the shoulder position.
(2) Get the elevation threshold $th_{e}\;$according to the relative elevation referring to the shoulder by (12).
(3) Determine the indexes$\;(i.e.,\;k_{1},k_{2},\ldots ,k_{N})$ of echo data from the upper body.
(4) Take the average of the corresponding azimuth positions$\;X(k_{i})\;(i=1,\;2,\ldots ,N)$ as the azimuth threshold $th_{a}$ as conducted in (13).

Finally, the MDRTs referring to different limbs can be categorized and extracted by using the thresholds as shown in Table 2.

We highlight the real-time implementation of our algorithm. As shown in Figure 5, the major time-consuming steps of our algorithm are the range compression and the slow-time Doppler processing, which can all be completed via the FFT. As for the CFAR step, it should not cause trouble for the current DSP chips [39]. All in all, the proposed algorithm is appropriate for real-time implementation, and it is not difficult.

In the following, the experiments carried out to validate the proposed approach are described; the motion components can not only be separated but also identified with the corresponding limbs.

## 4. Experimental Results

### 4.1. Experimental Setup

**Figure 7:**
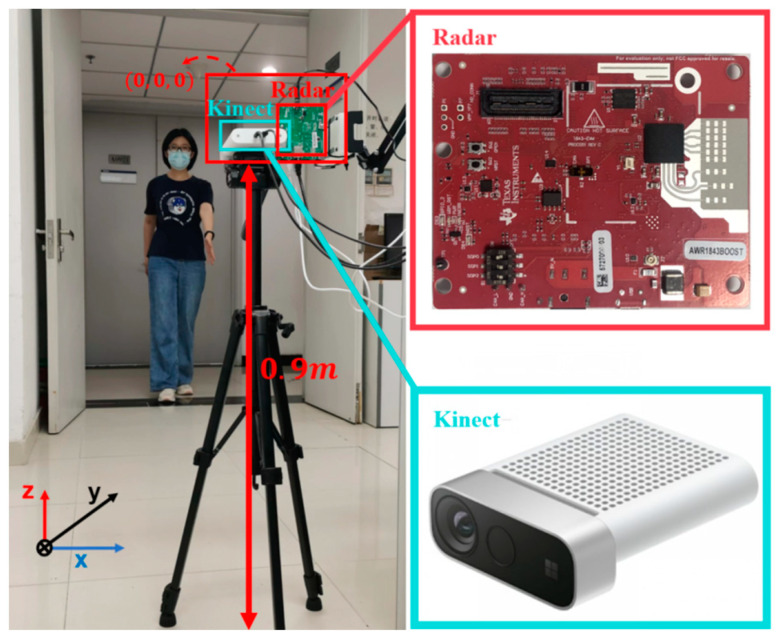
Experimental scene.

The experiment setup includes a radar demo board AWR1843 produced by Texas Instruments and a Kinect sensor developed by Microsoft as shown in Figure 7. The AWR1843 works at 77 GHz ($\lambda_{0}=3.896\;mm$) with a bandwidth of 4 GHz, which has three transmitting antennas and four receiving antennas. Here, only two transmitting antennas and two receiving antennas are configured to form the vertical and horizontal baselines with $d_{a}=d_{e}=\frac{1}{2}\lambda_{0}$, which is equivalent to the radar configuration of one transmitting three receiving (1T3R) as shown in Figure 4. The Kinect sensor can provide the motion data of human joints, which are used to validate the effectiveness of the proposed method.

Although the Kinect can just provide the distances of joints, their differentials can be calculated to obtain the velocities. We should mention that the output frame rate of the Kinect sensor is only 30 FPS, and the measurement is vulnerable to the environment variations (such as the light intensity and temperature). In addition, the skeleton tracking at the ends of the limbs reveals the greatest instability [40], especially at the hand joints [41]. As we know, fluctuations in the distance caused by skeleton tracking errors will induce more serious fluctuations in velocity. To mitigate these effects, a low pass filter is applied to Kinect data as preprocessing [42]. In the experimental scene, stable light and suitable temperature are kept to guarantee the quality of Kinect data.

Since the radar and the Kinect are very close to each other compared with the distance to the target as shown in Figure 7, they are supposed to be situated at the origin of the coordinates system, i.e., (0, 0, 0). And the ground is situated at z=−0.9 m. We describe three experiments that were conducted, i.e., swinging hands without moving, marking time, and walking. Three volunteers participated in the experiments, whose heights are listed in Table 3.

### 4.2. Experiment Swinging Arms

**Figure 8:**
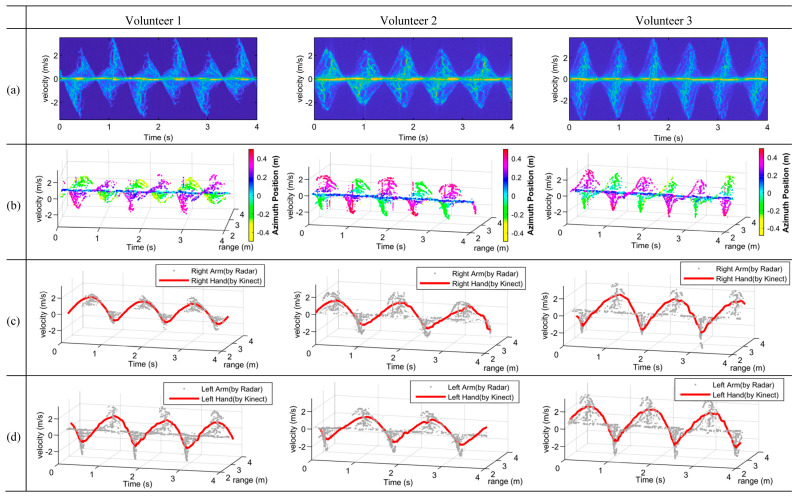
Experiment results of the swinging arms of three volunteers. (**a**) mDS of swinging arms. (**b**) mDRS of swinging arms with azimuth positions presented. (**c**,**d**) Extracted MDRTs of the right and left arms compared with the measured trajectories by Kinect, which are denoted by red lines.

In this experiment, the experimenter stood still on the ground and swung both arms with a cycle of about 1 s. The distance between the experimenter and the radar was about 2.5 m, i.e., the coordinates were (0, 2.5, −0.9). The experimental results are presented in Figure 8, where the results are grouped in different columns. Figure 8a shows the traditional mDSs of the three volunteers when swinging arms, Figure 8b shows the mDRSs of the swinging arms with the azimuth position information presented. As can be seen from Figure 8b, the overlaps of the multiple motion components exhibited in Figure 8a have been well eliminated by using the range information provided by the wideband radar.

Because only the arms were in motion in this experiment, the azimuth information is enough for discriminating between the right arm and the left arm. The azimuth position information is exhibited in Figure 8b as different colors, which was obtained from the azimuth interferometric phase according to (10), e.g., the green approximately represents the −0.3 m azimuth position, while the purple represents the 0.3 m azimuth position. The results were in accordance with the actual situation with the right arm at the negative azimuth position and the left arm at the positive azimuth position.

As shown in Figure 8b, the two arms’ mDRSs can be discriminated, their MDRTs can thus be extracted separately, as shown in Figure 8c,d, respectively, and finally, the motions of the left and right arms can be perfectly separated for all volunteers. As mentioned above, the Kinect tracks the human joints, while the radar detects the limbs. In fact, the end of each limb has the maximum radial speed for that limb; thus, the joints are extracted from the Kinect data because they correspond to the ends of the limbs, which are then used to match the envelop of the corresponding limbs. For instance, the hand joints of the Kinect data were extracted to match with the echo data of the arms. In this paper, the Kinect data were taken as an approximate truth value utilized to qualitatively evaluate the accuracy of the extracted limbs by radar, which are denoted by the red lines in Figure 8. As we can see from Figure 8c,d, the red lines were highly consistent with the envelops of the extracted positions.

### 4.3. Experiment on Marking Time

In this subsection, the experiments conducted on marking time with both the arms and the legs in motion are described, i.e., more micromotions will be involved and extracted. In this experiment, the volunteers still stood about 2.5 m away from the radar as before, i.e., their positions were (0, 2.5, −0.9). The motion time circle was about 1.5 s. Particularly, because the volunteers did not move forward or backward, their feet had zero radial velocity relative to both the radar and the Kinect; hence, the maximum Doppler frequencies of the legs were mainly induced by the knees.

**Figure 9:**
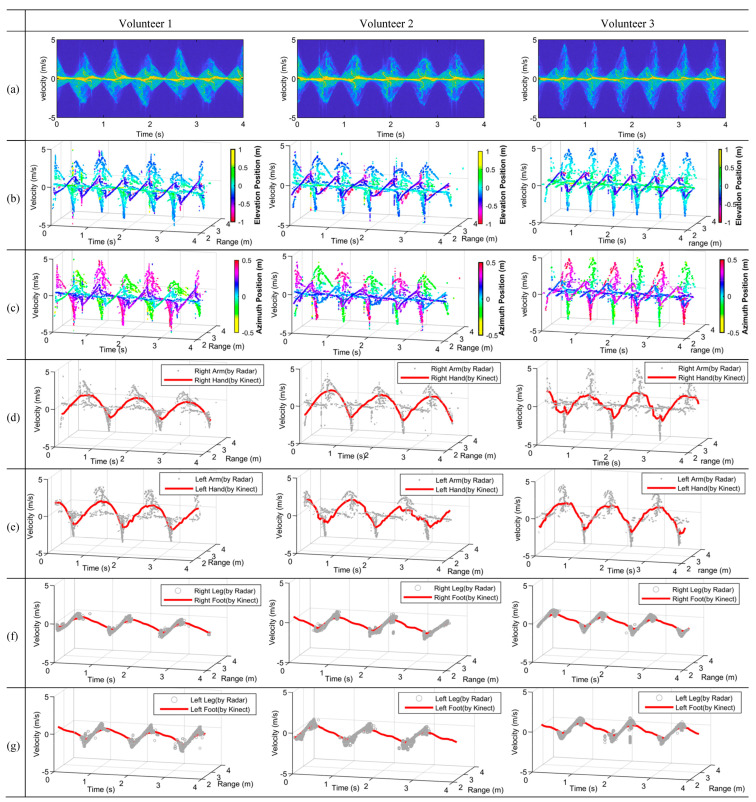
Experiment results on marking time for three volunteers. (**a**) mDS. (**b**) mDRS with elevation positions presented. (**c**) mDRS with azimuthal positions presented. (**d**–**g**) Extracted MDRTs of marking time compared with the measured trajectories by Kinect, which are denoted by red lines: (**d**) right arm, (**e**) left arm, (**f**) right leg, (**g**) left leg.

Figure 9a presents the mDSs of the three volunteers when marking time; it is clearly shown that the mDSs of the different limbs overlap. Figure 9b presents the mDRSs of marking time with the elevation position information presented, while Figure 9c presents the mDRSs with the azimuth position information presented. As can be seen from Figure 9b, the mDRSs were more complicated than that of the swinging arms. It is a more challenging task to discriminate and extract the micromotions of arms and legs.

As shown in Figure 9b, the light blue trajectories cover the largest elevation range, i.e., they belong to the swing arms. The dark blue and purple trajectories correspond to the left and right legs according to their elevation positions. In Figure 9c, there are mainly two motion types differentiable according to the azimuth position information, i.e., the purple color denotes the motions of the right limbs including the right hand and the right leg, while the green color denotes the motions of the left limbs including the left hand and the left leg.

The extracted results of the marking time experiments of the three volunteers are presented in Figure 9d–g. As shown in Figure 9d,e the motions of the right and left arms have been perfectly separated, and it is also true for the motions of the right and left legs, as shown in Figure 9f,g. The hand and knee joints were extracted from the Kinect data, which are presented by red lines in Figure 9d–g. The MDRTs of the arms and legs extracted by radar all agreed very well with the trajectories of the Kinect.

### 4.4. Experiment on Walking

In the last experiment, micromotions of walking human were extracted, which are much more complicated than that of the previous two experiments because the arms and legs induce micro-Doppler frequencies that are significantly larger than before, resulting in much more serious overlapping problems. During the experiment, the volunteers walked away from the radar from about 1.5 m to 4.0 m at a speed of around 1 m/s, i.e., walked from (0, 1.5 m, −0.9 m) to (0, 4.0 m, −0.9 m). In this case, the speed of the feet was greater than that of the knees.

**Figure 10:**
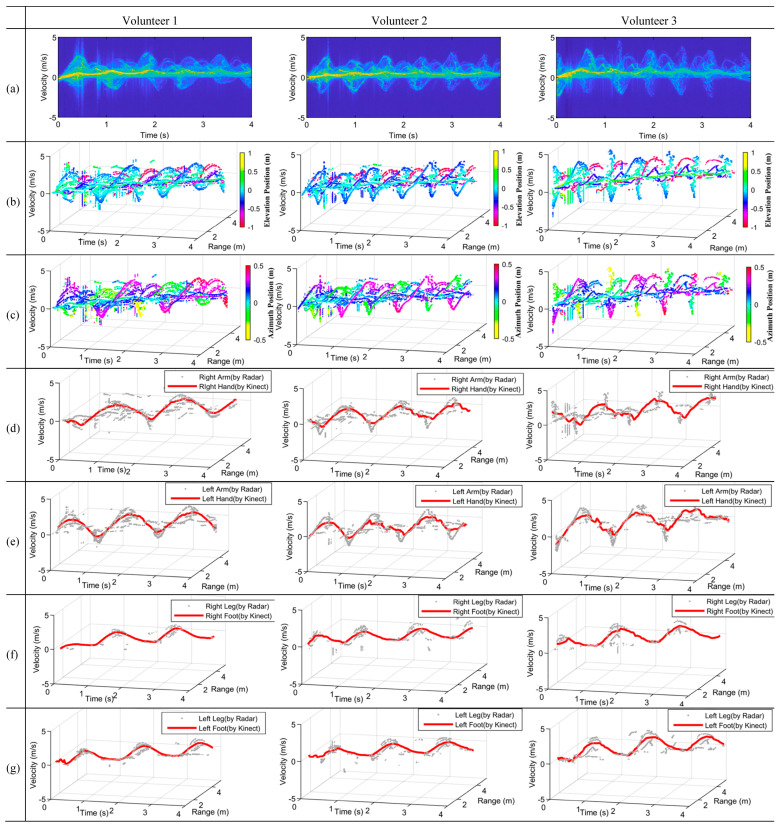
Experiment results on walking for three volunteers. (**a**) mDS of walking. (**b**) mDRS with elevation positions presented. (**c**) mDRS with azimuthal positions presented. (**d**–**g**) Extracted MDRTs compared with the measured trajectories by Kinect, which are denoted by red lines: (**d**) right arm, (**e**) left arm, (**f**) right leg, (**g**) left leg.

The experimental results are presented in Figure 10. As can be seen from Figure 10a, serious overlaps are exhibited in the traditional mDS images as before, although the body induced a different shape. The information of the elevation position and azimuth position corresponding to different limbs is provided along with the mDRSs in Figure 10b,c, respectively. Compared with Figure 9b, the purple color in Figure 10b is much more obvious, and the denoted position is about −0.8 m, i.e., the corresponding velocities refer to the feet. Figure 10d–g present the extracted MDRTs of different limbs, from which one can see that the right arms and legs and the left arms and legs have all been separated very well for all three volunteers, and all are in good accordance with the results of the Kinect.

Last but not least, one may consider the scenarios with multiple human targets. Generally speaking, clustering and tracking steps may be required before limb separation, and more powerful radar with more channels can be applied to cope with this situation. We should emphasize that if multiple human targets are covered by the radar beam at the same time and they can be resolved in range, then the proposed approach still can be applied to extracting the limbs of different humans.

## 5. Conclusions

In this paper, a novel method is proposed for extracting micromotions of human limbs based on the MDRTs retrieved by using wideband interferometric radar, which has been configured as 1T3R mode, forming orthogonal interferometric baselines along the vertical and the horizontal directions. In this approach, the range information is first incorporated into the traditional mDS to eliminate the overlaps of different limbs. Then, the azimuth and elevation positions of the limbs are determined by utilizing the interferometric phases. Three experiments on swinging arms, marking time, and walking by three different volunteers were carried out, using a commercial off-the-shelf radar module. At the same time, a Kinect sensor was used to simultaneously record the micromotions to verify our experiment results. The proposed method integrated the time, Doppler, and range information altogether, to realize the time-Doppler-range 3D motion data extraction of human limbs using the obtained interferometric position information.

All the experiments demonstrated that the overlapping problems were solved very well. A wide prospect of applications of the proposed approach can be envisaged, e.g., surveillance of human activities, health care monitoring, human identification, as well as human–computer interaction. Future work will focus on human motion recognition and classification based on the extracted micromotions and based on which adaptive joints’ extraction can be realized. We should point out that the challenge of extracting the left leg and the right leg should be considered, if the two legs move in a line or they are too close to each other in azimuth. So, it remains an open problem requiring further experiments using more powerful radar configuration, for instance, multistatic radar is expected to obtain more reliable and more robust extraction of human limbs and joints.

## Figures and Tables

**Table 1:** Comparison of related works.

Ref.	Tx/Rx Channels	Angle Information	Object	Aim	Data Represented Domain	Operating Freq.	Year
[25]	1 × 3	Azimuth and Elevation	Moving humans	Multiple humans tracking	Azimuth elevation range domain	$f_{1\;}$= 2.4 GHz $f_{2}\;$= 2.39 GHz	2006
[28]	1 × 3	Azimuth and Elevation	Two pedestrians	Image separation	Azimuth elevation range domain	$f_{0\;}$= 26.4 GHz w = 500 MHz	2014
[31]	1 × 10	Azimuth	Human limbs	Extraction of limbs	Time-Doppler domain	$f_{0}\;$= 77 GHz, w = 2 GHz	2019
[2]	1 × 3	Azimuth and Elevation	Human hands	Gesture recognition	Range-Doppler map interferometry map	$f_{0}\;$= 60.75 GHz w = 4.5 GHz	2021
[32]	16 (Rx)	Azimuth and Elevation	Pedestrian	Feet tracking	Time-Doppler range domain	$f_{0\;}$= 76.5 w = 1 GHz	2022
[this work]	1 × 3	Azimuth and Elevation	Human limbs	Extraction of limbs	Time-Doppler range domain	$f_{0\;}$= 79 GHz, w = 4 GHz	2023

**Table 2:** Classification of limbs by thresholds.

	Azimuth PositionX	Elevation Position Z
Right Arm	$X<th_{a}$	$Z>th_{e}$
Left Arm	$X>th_{a}$	$Z>th_{e}$
Right Leg	$X<th_{a}$	$Z<th_{e}$
Left Leg	$X>th_{a}$	$Z<th_{e}$

**Table 3:** Heights of the three volunteers.

	Volunteer 1	Volunteer 2	Volunteer 3
Height	176 cm	166 cm	170 cm

## Data Availability

Not applicable.
